# Preface, MSM 2011

**Published:** 2011

Brain, Mind and Consciousness: An Interdisciplinary International Perspective

The *Mens Sana Monographs,* along with the World Psychiatric Association [Philosophy and Humanities Section] and the Indian Council of Philosophical Research, co-sponsored an International Seminar on Mind, Brain and Consciousness. It was conducted by VPM´s Joshi-Bedekar College of Arts and Commerce, at its campus at Thane, Maharashtra, India, 13 to 15 January, 2010. *MSM 2011,* titled, *Brain, Mind and Consciousness: An Interdisciplinary International Perspective,* is based on the papers presented there, which have been peer-reviewed and substantially revised to conform to *MSM* guidelines and standards. It has contributions from philosophers, psychiatrists, psychologists, neurosurgeons and other neuroscientists, even an anthropologist.

The Editors of *MSM,* Ajai R. Singh, Shakuntala A. Singh, write an extended editorial: Brain-Mind Dyad, Human Experience, the Consciousness Tetrad and Lattice of Mental Operations: And, Further, The Need to Integrate Knowledge from Diverse Disciplines [p6].

Nancy C. Andreasen, MD, PhD, Andrew H. Woods Chair of Psychiatry at University of Iowa Carver College of Medicine, USA, and Past Editor, *American Journal of Psychiatry* [1992-2005] writes on, ´A Journey into Chaos: Creativity and the Unconscious´ [p42]

R. Balasubramanian, Chairman, Indian Philosophical Congress and President, Afro-Asian Philosophy Association writes a paper, ´Consciousness, Cognition and the Cognitive Apparatus in the Vedānta Tradition´ [p54].

K.W.M. Fulford, Dphil, FRCP, FRCPsych, Fellow of St Cross College, Member of the Philosophy Faculty and Honorary Consultant Psychiatrist, University of Oxford; Emeritus Professor of Philosophy and Mental Health, University of Warwick; Editor, *Philosophy, Psychiatry, and Psychology* and Special Advisor for Values-Based Practice, Department of Health, London, writes a paper, ´Neuroscience and values: a case study illustrating developments in policy, training and research in the UK and internationally´ [p79].

Donelson E. Dulany, Professor of Psychology Emeritus at the University of Illinois and Past Editor [1988-2009], *American Journal of Psychology*, writes on, ‘What Should Be the Roles of Conscious States and Brain States in Theories of Mental Activity?´ [p93].

George E. Vaillant, Professor of Psychiatry at Harvard Medical School and the Department of Psychiatry, Brigham and Women´s Hospital, writes on,´ The Neuro-Endocrine System And Stress, Emotions, Thoughts And Feelings´ [p113].

Sunil K. Pandya, M.S., Neurosurgeon, Jaslok Hospital and Research Centre, Mumbai, India, writes a paper on, ´Understanding Brain, Mind and Soul: Contributions from Neurology and Neurosurgery´ [p129].

William Hirstein, Chair, Philosophy Department, Elmhurst College, Illinois, USA, writes a paper on, ´The Contribution of Prefrontal Executive Processes to Creating a Sense of Self´ [p150].

Horacio Fabrega Jr., Professor of Psychiatry and Anthropology, University of Pittsburgh School of Medicine, Western Psychiatric Institute and Clinic, Pittsburgh, USA., writes on, ´Sickness and Healing and the Evolutionary Foundations of Mind and Minding´ [p159].

Alfredo Pereira Jr., Adjunct Professor on Philosophy of Science, Institute of Biosciences, State University of São Paulo (UNESP), Campus Rubião Jr., Botucatu-SP, Brasil; Maria Alice Ornellas Pereira, Adjunct Professor on Psychiatric Nursing, Faculty of Medicine, State University of São Paulo (UNESP), Botucatu-SP and Fábio Augusto Furlan, Professor of Physiology, Faculty of Medicine and Nursing, University of Marília, Marília-SP, write a paper on, ´Recent Advances in Brain Physiology and Cognitive Processing´ [p183].

Christian Perring, Professor of Philosophy at Dowling College, New York, USA, and Editor, *Metapsychology Online Reviews*, writes a paper on, ´Bridging the Gap between Philosophers of Mind and Brain Researchers: The Example of Addiction´ [p193].

Neeta Mehta, PhD, Associate Professor, Department of Psychology, KET´s V. G. Vaze College, Mumbai, India, writes on, ´Mind-Body Dualism: A Critique from a Health Perspective´ [p202].

Avinash De Sousa, MD, DPM, DPC, DHA, MBA (Human Resource Development), MSc (Psychotherapy and Counselling), Consultant Psychiatrist and Psychotherapist, Mumbai, India, writes a paper, ´Freudian Theory and Consciousness: A Conceptual Analysis´ [p210].

Manoj Patharkar, Assistant Professor, Joshi Bedekar College, Thane, India, writes a paper on, ´From Data Processing to Mental Organs: An Interdisciplinary Path to Cognitive Neuroscience´ [p218].

O. Olugbile, FRCPsych., and M.P. Zachariah, PhD, Department of Psychiatry, Lagos State University Teaching Hospital, Lagos State, Nigeria, write a paper on, ´The Relationship between Creativity and Mental Disorder in an African Setting´ [p225].

Daniel A. Drubach, Alejandro A. Rabinstein, Department of Neurology, Mayo Clinic, Rochester, Minnesota, USA; and Jennifer Molano, Department of Neurology, University of Cincinnati, Ohio, USA, write a paper on, ´Free Will, Freedom of Choice and Fronto-temporal Lobar Degeneration´ [p238].

Gabriel José Corrêa Mograbi, Professor of Philosophy of Science, Philosophy of Mind and Epistemology at Federal University of Mato Grosso, writes on, ´Neural Basis Of Decision-Making And Assessment: Issues On Testability And Philosophical Relevance´ [p251].

Nilanjan Das, Graduate Student, Department of Philosophy, Jadavpur University, Kolkata, India, writes on, ´The Concept of Thinking: A Reappraisal of Ryle´s Work [p260]

Namita Nimbalkar, PhD, Head, Department of Philosophy; Director, Gandhian Studies Centre, Birla College, Kalyan, Maharashtra, India, writes a paper, ´John Locke on Personal Identity´ [p268].

Gabriel José Corrêa Mograbi, Professor of Philosophy of Science, Philosophy of Mind and Epistemology at Federal University Of Mato Grosso, writes another paper, ´Meditation And The Brain: Attention, Control And Emotion´ [p276].

´ A Discussion in the Mind_Brain_Consciousness Group: Let´s Study the Structure that is the Very *Raison de etre* of Our Existence´ is a recent discussion in which Ajai Singh, Richard Godwin, Gabriel Mograbi, R. Balsubramanian, Veena Garyali participate [p284].

Ajai R. Singh, Editor, MSM, writes in journalology, ´Science, Names Giving and Names Calling: Change NDM-1 to PCM´ [p294]. He also writes, ´Template for MSM Submissions,´ a guide to writers/researchers who wish to submit their writings to MSM [p320].

The Journal puts out a Call for Papers for MSM 2012 Theme Monograph on *Mind, Consciousness and the Brain: Contributions from Indian Thought* [p324].

Some other information to share:

1. Prof Nancy C. Andreasen MD, PhD, Past Editor, *American Journal of Psychiatry*; Andrew H. Woods Chair of Psychiatry and Director of its Neuroimaging Research Center and the Mental Health Clinical Research Center, University of Iowa Carver College of Medicine, Iowa City, IA, has joined the Honorary International Advisory Board of *MSM* from 2 Jan 2010. MSM has great pleasure in welcoming her to the Board. [Available at:http://www.msmonographs.org/aboutus.asp]
2. Kindly do spare some time to see the various features at the Journal website www.msmonographs.org. You can view archives at http://www.msmonographs.org/backissues.asp, Most popular articles at http://www.msmonographs.org/showstats.asp?a=ts, Most cited articles at http://www.msmonographs.org/showstats.asp?a=tc. All articles now have a DOI, and are available HTML Full text, PDF, Mobile Full Text, Epub, and Sword Plug in for Repository. Medknow Publications is doing a great job of maintaining the website and publishing the Journal.
3. *MSM* always had some special features, like Authors´ bios with their photograph, and ´Questions that this paper raises.´ An abstract at the beginning, and concluding remarks and take home message at the end were made for ease of comprehension for readers, and for precision in authors. Since 2010, authors have to also submit a flowchart of their papers, a pictorial representation of how the thought flows through their writing. This is to add further clarity and precision to write-ups and ease of comprehension for readers. Also, authors are strongly recommended to quote recent works, and at least 50% of their references are expected to be 2000 and later. This is to make write-ups as contemporary as possible.
4. *MSM* features in the Wikipedia at http://en.wikipedia.org/wiki/Mens_Sana_Monographs.
5. The *MSM* 2010 editorial “Modern Medicine: Towards Prevention, Cure, Well-being and Longevity” Available at http://www.msmonographs.org/article.asp?issn=0973-1229;year=2010;volume=8;issue=1;spage=17;epage=29;aulast=Singh] has been translated in Portuguese and republished in June 2010 by *Revista Latinoamericana de Psicopatologia Fundamental*, with permission, as “Medicina moderna: rumo à prevenÇão, à cura, ao bem-estar e à longevidade” [Available at http://www.scielo.br/scielo.php?script=sci_arttext&pid=S1415-47142010000200008&lng=en&nrm=iso&tlng=pt
6. *MSM* 2008 titled, ´Medicine, Mental Health, Science, Religion and Well-being´ has been reviewed in 2010 by *Metapsychology Online Reviews* Available at http://metapsychology.mentalhelp.net/poc/view_doc.php?type=book&id=5379&cn=394]
7. *MSM* 2006 has been earlier reviewed by the *Indian Journal of Psychiatry* Available at http://www.indianjpsychiatry.org/article.asp?issn=0019-5545;year=2007;volume=49;issue=1;spage=71;epage=72;aulast=Bhide]
8. An earlier *MSM* 2006 editorial, ´What is a good editorial?´ Available at http://www.msmonographs.org/article.asp?issn=0973-1229;year=2006;volume=4;issue=1;spage=14;epage=17;aulast=Singh] had been republished, with permission, as an editorial in *Indian Journal of Pharmacology* Available at http://www.ijp-online.com/article.asp?issn=0253-7613;year=2006;volume=38;issue=6;spage=381;epage=382;aulast=Singh]

As we go to press, our publisher informs us that MSM is now indexed with PubMed and PubMed Central. See http://www.ncbi.nlm.nih.gov/sites/entrez?db=pubmed&term=%22Mens%20Sana%20Monogr%22%5Bjour%5D and [http://www.ncbi.nlm.nih.gov/pmc/issues/193894]. This is really heartening news. We thank all our authors, our Honorary International Editorial Board Members, our Board of Peer Reviewers, our Subscribers, our Publishers (Medknow Publications) and our readers and supporters who stood by us all through. We take this opportunity to further our commitment to the understanding of ´medicine, mental health, mind, man and their matrix´.

We also thank all those who have sent congratulatory messages and called to convey their good wishes.

It all makes it seems so worthwhile. Hope you continue with us all the way

The Editors, MSM

mensanamonographs@yahoo.co.uk

30 January 2011.

